# Evaluation of Chute Exit, Novelty and Human Approach Tests in Mertolenga Beef Cattle

**DOI:** 10.3390/ani13061087

**Published:** 2023-03-18

**Authors:** Andreia Vitorino, George Stilwell, José Pais, Nuno Carolino

**Affiliations:** 1 INIAV—Instituto Nacional de Investigação Agrária e Veterinária, I.P., Fonte Boa, 2005-048 Vale de Santarém, Portugal; 2 CIISA—Centre for Interdisciplinary Research in Animal Health, Faculty of Veterinary Medicine, University of Lisbon, Av. Universidade Técnica, 1300-477 Lisboa, Portugal; 3 Associate Laboratory for Animal and Veterinary Sciences (AL4AnimalS), 1300-477 Lisboa, Portugal; 4 ACBM—Associação de Criadores de Bovinos Mertolengos, Rua Diana de Liz Horta do Bispo, Apartado 466, 7006-806 Évora, Portugal; 5 Escola Universitária Vasco da Gama, Av. José R. Sousa Fernandes 197, Lordemão, 3020-210 Coimbra, Portugal

**Keywords:** behavior, selection, genetics, animal welfare, animal handling

## Abstract

**Simple Summary:**

The increase in farms’ size over time while not increasing livestock density, and the extension of the area where animals are kept, has meant that the proximity between human and animals has decreased. More than ever, good management is essential, as it is associated with better performances and overall welfare improvement. The personality traits most commonly studied in farm animals are exploration and sociability, and are usually studied by observing animals’ behavior. The present work aimed to evaluate the behavior of Mertolenga breed young bulls when exiting the chute, the reaction to novelty and human approach tests, as well as to understand if the responses between these tests are related. Data from twenty-nine Mertolenga-bred young bulls was collected and analyzed, and it was observed that older animals showed a tendency to leave the chute more calmly, took longer to touch the novelty ball and touched the ball less often, with a consequent reduction in the likelihood of playing with it. As for the human approach test, animals that came out of the chute more calmly allowed the human to come closer to them. These behavior tests should be further studied on potential sires in order to increase docility and handling in autochthonous beef breeds.

**Abstract:**

Livestock behavior and welfare are increasingly recognized to be related, not only to the animals’ handling, but also with productivity. The present work was carried out at the Mertolenga Breed Testing Center and its objective was to evaluate the behavior of Mertolenga breed young bulls when exiting the chute, the reaction to novelty and human approach tests, as well as to understand if the responses between these tests are related. Twenty-nine Mertolenga-bred young bulls from 16 different farms, aged between 8 and 13 months, entered the study farm from the end of May to the beginning of June 2021. Data was collected on six different days and analyzed with the SAS^®^ 9.4 software. Older animals showed a tendency to leave the chute more calmly, take longer to touch the novelty ball and to touch the ball less often, with a consequent reduction in the likelihood of playing with it. In the human approach test, animals that came out of the chute more calmly allowed the human to come closer. These behavior tests should be further studied on potential sires, so as to increase docility and manageability of autochthonous beef breeds.

## 1. Introduction

Although the number of beef cattle farms has been decreasing, the stock numbers have been increasing. The increase in population size and the extension of the area where they are kept has reduced the proximity between humans and animals, with the possibility that the brief interactions are limited to unpleasant procedures for the animal, making them afraid of humans and the handling of the animals more dangerous for the operator.

The intrinsic characteristics of animals such as genetics, past experiences, and the context they are in, as well as the attitude of humans themselves, will influence their relationship with the production environment [1]. The importance of human-animal relationship has been intensely studied, and its influence on the welfare, health, and performance of cattle is clear. While bad animal handling can lead to production declines due to increased fear and consequent release of stress hormones, good handling can improve performance and animals’ overall welfare [2]. Animals’ reactivity results not only from a set of experiences throughout their lives, but also from genetic factors [3]. Table 1 shows heritability estimates for some behavioral tests in different cattle breeds.

There are several methods for temperament assessment that can be used in cattle: from measurements of physiological parameters, such as heart rate or blood cortisol level, to behavioral tests, such as scoring behavior and speed when exiting a chute, the flight distance, and the novelty test, among others.

The most studied personality traits in production animals are boldness (defined as the propensity to take risks when exposed to novelty), exploration (behavior by which animals gain knowledge about their environment), and sociability (the propensity to seek contact with others or stay close to conspecifics), and are usually studied by observing animals’ behavior in novelty or isolation tests [8]. Ensuring calmness in animals is also a good strategy to control feeding behaviors, as well as overall activity. Thus, with longer and more frequent meals, feed efficiency and methane emissions are improved [9]. Furthermore, selecting and breeding less temperamental and fearful animals ensures their welfare and makes the production sector more attractive to consumers.

The Mertolenga breed is the most numerous out of the 15 autochthonous cattle breeds, currently with about 27,000 breeding females in Portugal. It presents an excellent adaptability and three different phenotypes: red, roan, and red spotted [10]. It is widely known for its nervous temperament that sometimes makes it challenging to work with.

Cattle, as preys, are very prone to fear, which causes their responses to stimuli to be unpredictable. Thus, fear is a very important characteristic that often affects welfare in a negative way. According to [11], fear is an emotion induced by a perceived threat that causes animals to quickly move away from its location or to hide. It should be distinguished from emotional anxiety, which typically occurs without a particular or immediate external threat. Some examples of signs of fear are running away, seeking to hide, an exaggerated reaction upon exposure to a sudden novelty, vocalizations, conspecific seeking, and also increased aggression, including attacking the source of fear. The reactivity of cattle to handling and to human contact are consistent over time and may have some genetic influence, thus, temperament traits are already used in some selection programs. However, the diversity of behavioral tests makes it difficult to demonstrate a relationship between them [12].

As cattle are gregarious animals, fear expression can also be influenced by social stimuli, and social isolation is probably one of the most important stressors when animals perform fear tests individually. Thus, social isolation may act as a multiplying factor in the ability to feel fear, especially neophobia. Behavior patterns revealing fear are very variable according to the characteristics of the threat, and can be expressive movements, such as head posture or facial expressions. There are also some specific alarm signals, such as odors or pheromones, which allow herbivores to communicate and warn conspecifics [13].

Fear tests should be performed when the animals are still young, in order to minimize the influence coming from previous experiences. The results of these tests can not only give us information about the temperament of the animal, but may also give indications about the future productivity of the tested animal [14]. Both the novelty test and the human approach test are often used in animal fear studies.

The novelty test, such as the evaluation of the animal’s reaction to a new object, allows us to identify the animal’s confidence, its tendency to explore (as an indicator of its daringness), whether it is ready to take risks, and its adaptability to new environmental conditions, which may affect its well-being and productivity. Animals’ response to a novel object also reflects its degree of fear [15]. This test is usually performed outside the animal’s comfort environment and can be repeated several times in order to study whether the behavior toward the object is maintained or changes over time, reflecting an adaptive capacity.

The response to a new object depends on the experimental design, its validity, and its reliability. The repeatability of the test is often questioned, since habituation is to be expected [13]. In case of repeating the test, it should preferably be performed in the same place, with the animal alone. The insertion of a new object in the space will cause abruptness, unfamiliarity, and unpredictability factors to produce (or at least affect) animals’ reactions. The stimuli tested are mostly visual, with a person introducing an object on the floor before the animal’s habituation to the site, or with an object being dropped suddenly near the animal. The habituation period to the test site before confrontation with the new object, can vary from 1 to 15 min, and the animals tested should be calves or young bulls, in order to minimize the impact of previous experiences on their behavior [14]. The most commonly used variables are the latency to approach the object, the minimum distance to the object, the frequency and duration of contact, the type of exploration (e.g., licking, sniffing), body posture, and vocalizations.

In a study by [8] almost all of the behaviors studied in the novelty test with an object before and after weaning—such as the latency to touch, the percentage of time focused or not focused, the percentage of time the animal touched the object, and the number of times it played with the ball—tended to be correlated.

Other behavior tests are possible when handling animals in a race or chute, including the type and speed of exiting. The exit from the chute has been used as a measure to evaluate temperament and is indicative of the same type of stress that animals suffer when they meet humans. Cortisol levels are positively correlated with altered behavior inside the chute and with speed when exiting it. These behaviors reflect higher stress levels and are usually associated with less time feeding and resting, and with a compromised immune system [16].

The exit velocity from the chute can be evaluated using infrared sensors 1.83 m away from the chute according to [17]. This is a simple method to implement and is safe for the operators performing it, while also being simpler for the animals, as they do not have to be further restrained or be manipulated, as in the test where the percentage of eye-white or the behavior of the animal locked in the chute is determined [18]. A study showed that the exit speed from the chute tended to increase with age [18].

The human approach test is more easily performed in the animal’s comfort environment and assesses the human-animal relationship (in this case, fear from humans) which impacts the safety of stockpersons, as well as the well-being and productivity of the animals [15].

The quality of the human-animal relationship depends on the behavior of both parties. However, animals usually generalize attitudes and behaviors toward humans (although cattle can discriminate between different people). The interactions between the two can be subdivided into five main types: static visual presence, walking among animals without tactile contact (but with the possibility of using their voice), physical contact, feeding, and intrusive [19]. The animal can perceive whether an interaction is positive, neutral, or negative, and this perception is in turn related to the animal’s previous experiences. Humans can unconsciously emit calm or dangerous signals, often ignoring fear, aggression, or calm signals from the animal, therefore human behavior and know-how is crucial. It is humans who mostly determine the number and nature of their contact with animals, with animals only reacting to them (although they may also initiate them). Some studies show that neither the breed nor the age of the animals significantly influences the animals’ withdrawal reactions, but rather the human-animal relationship existing on the farm. The size of the herd also has an effect on the animals’ reactions, with animals coming from larger herds being calmer [2].

The objective of the present work was to evaluate the behavior of Mertolenga-breed young bulls when exiting the chute, when exposed to novelty, and in a human approach test, as well as to understand whether the responses between these tests are related. The results may support the inclusion of some of these tests in the classification of bulls to be used as breeders.

## 2. Materials and Methods

### 2.1. Facilities, Animals and Management

This study was conducted at the Mertolenga Cattle Breed Testing Center in Évora (Portugal) between May and November 2021.

The Mertolenga Testing Center is capable of testing up to 100 males simultaneously. The performance tests carried out aim to contribute to breeding programs, and to identify and select the best bulls to be used as semen donors for use in the breed’s artificial insemination program, and for storage in the Portuguese Bank of Animal Germplasm.

The males to be tested are chosen according to their genetic merit for calving interval and maternal ability, their development, and their conformation. The performance tests follow a protocol approved by the Portuguese competent authority (Direção Geral de Alimentação e Veterinária—DGAV) and in accordance with the standards of the International Committee for Animal Recording. Upon entering the center, the animals undergo a 23-day adaptation period to individual feeders, after which the performance test begins. At the end of the testing period, the males that pass the technical evaluation, are morphologically classified and submitted to an andrological examination. Only the approved males whose andrological examination results are considered apt, are validated and registered as future breeding stock [20].

In this study, 29 Mertolenga young bulls entered the farm from the end of May to the beginning of June 2021. These animals came from 16 different farms, as described in Table 2.

As there was not enough room for all of the animals in the same paddock, they were subsequently divided into two groups according to their weights and were always kept in side-by-side roofed pens, with access to an outside loafing area. Although they were in two adjoining pens, the study was conducted as a single group.

Every three weeks, the animals were taken to the chute to be weighed in order to calculate their weight gains and to perform individual novelty tests. The chute was located on the side of the barn where the animals lived, along which there was an access corridor.

All tests were performed by the same person, dressed in the same way, in order to reduce possible influences.

### 2.2. Evaluation of the Chute Exit

Ideally, as mentioned, this test is performed using an infrared device for better accuracy. In this work, since it was not possible to use this type of equipment, we used the evaluation of the exit of the chute on a scale of 1 to 3, where 1 corresponds to an exit at pace, 2 at trot, and 3 at gallop.

**Figure 1:**
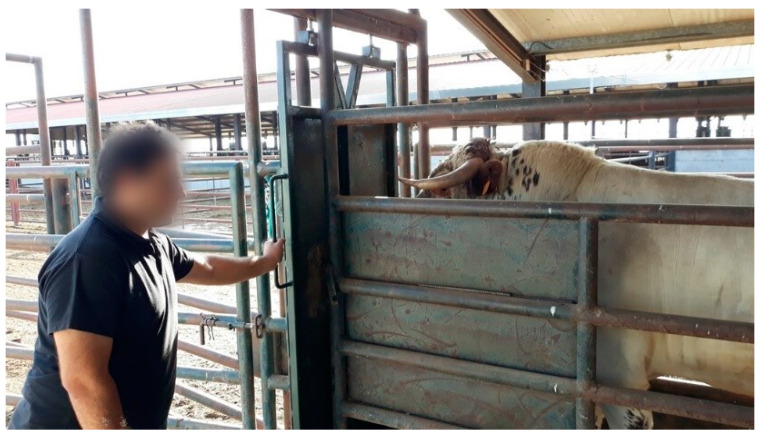
Evaluation of the chute exit [21].

This evaluation was always performed by the same operator, in order to minimize human error (Figure 1).

### 2.3. Novelty Test with a Ball

**Figure 2:**
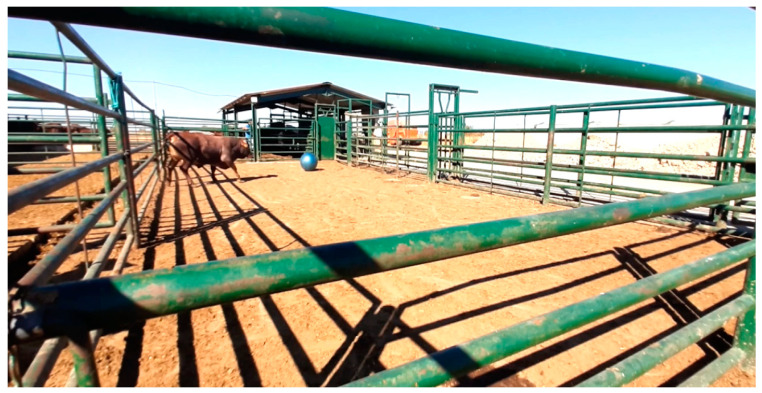
Novelty test with a ball.

Taking advantage of the fact that the animals were going to the chute to be weighed, a novelty test was performed with a striking-blue colored ball, of approximately 70 cm in diameter, placed in a fenced area adjacent to the chute (Figure 2). Each animal leaving the chute had to pass through this area, consisting of approximately 50 m^2^ earthen floor, which then opened to their usual home pen. In a first approach, only the behavior of the animal passing through this route was recorded. After 3 weeks, at the time of the new weighing, each animal had 1 min to become acquainted to the area before the ball was placed as a novelty factor for another 4 min. During this time, all behaviors were continuously recorded (e.g., vocalizations, defecations, time, and minimum distance of approaches to the ball, among others).

### 2.4. Human Approach Test at the Feed Corridor

The third test in this study, the human approach test, was conducted from the feeding corridor and the same animals were included so that the results of the different tests could be correlated. After the novelty test was performed, the animals remained in a pen near the chute, and only after all of the animals had performed the novelty test and the food distributed, were they returned to their holding place where the human approach test was performed.

**Figure 3:**
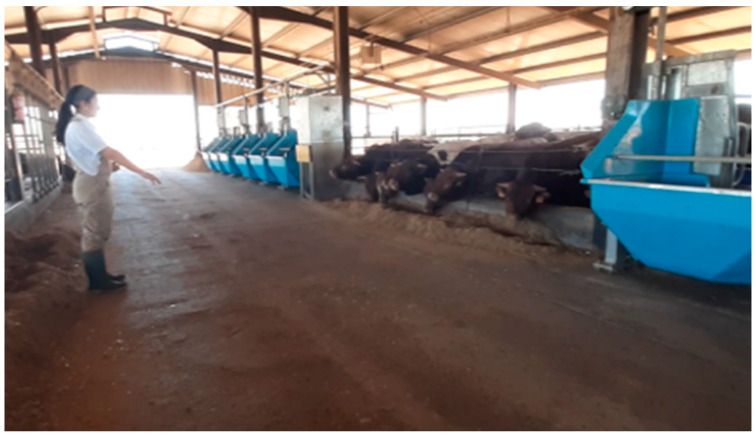
Human approach test at the feed corridor.

At 3 m away from the feed trough, when an animal was ideally isolated (with no more than two animals within 1 m of it), the operator began the approach by taking one step per second with one arm stretched forward at 45° and the palm facing down toward the animal (Figure 3). When the animal reacted and moved away or turned its face away, the test was terminated and the distance to the trough was recorded.

A scale of 0 to 3 was devised to assess the flight distance, with 0 corresponding to touching the animal, 1 to a distance of up to 1 m, 2 to a distance between 1 and 2 m, and 3 to a distance between 2 and 3 m.

### 2.5. Data Collection

All tests were filmed with a cell phone camera in order to facilitate subsequent analysis, and all field records were made on paper and later entered into an Excel sheet.

The records collected in the chute were the identification of the operator and the animal, the weight, and the type of exit from the chute (evaluated as pace, trot, or gallop).

In the novelty test, in the first 60 s in which the animal is alone in the pen adjacent to the chute, the following data were recorded: the time (in seconds) in which it was active or stopped, whether it moved toward the camera (located outside the pen) or to the water trough (present in the pen), and whether it vocalized. In the next 4 min the following were recorded: the latency time to touch the ball, the time the animal was attentive to the ball and distracted, the number of touches it gave the ball, whether or not it played with the ball (rated yes—1/no—0), the time it was active or immobile, whether it urinated/defecated, and again, whether it vocalized, went to the camera, or to the water trough.

When necessary, comments were added such as whether a person or tractor passed close to the pen during the test, among others.

Most of these records were collected directly onto Excel tables during the analysis of the test videos.

In the human approach test in the feeding corridor, the final distance from the operator to the feeding trough was calculated in a 0 to 3 scale, recorded on paper, and later confirmed by video analysis.

### 2.6. Statistical Analysis

For the statistical analysis, data collected on six different days during the performance test were used. In addition to the behavioral characteristics evaluated, the following information about each animal was considered: identification of the animal, date of birth, farm of origin (classified by letters in order to maintain confidentiality), and weight and age at the entrance and exit of the performance test. As mentioned earlier, some nominal variables were converted into numeric ones, namely the exit from the chute (pace—1, trot—2, gallop—3) and the playing behavior (yes—1, no—0). All data were collected in Excel tables.

All analyses were conducted with the SAS^®^ 9.4 software [22] using different procedures (Proc’s) with different models and including several variables as factors, but in the final model analysis of each behavior parameter, only the factors that significantly influenced them for *p* < 0.05 were considered.

Initially, several descriptive statistics were estimated with the Proc Means, Proc Freq, and Proc Univariate procedures in order to characterize the available data. A preliminary correlation analysis was also performed among all behavioral parameters to assess their associations, and then each parameter was analyzed individually.

Through the novelty test, we evaluated the exit from the chute (classified as pace, trot, or gallop), the activity of the animal, the latency to touch the ball (in seconds), the number of touches to the ball, and whether the animal played with the ball.

Chute exit was analyzed using Proc Glimmix, in which the effect of the farm of origin, age, and weight of the animal were included as factors. Farms that had only one animal (total of 5 farms) were removed from this analysis.

Activity behavior was initially analyzed using Proc GLM with a model that included the effect of the animal’s farm of origin, and later in another way, with a mixed model using Proc Mixed, in which the effects of the type of exit from the chute, the operator, and the animal were considered (the latter as a random effect).

The latency to touch was analyzed using Proc GLM with a model that included the effect of the farm of origin, and subsequently with a mixed model using Proc Mixed with a model that included the type of chute exit as a fixed effect, and the animal as a random effect.

The number of touches on the ball was also analyzed with a mixed model and with Proc Mixed, but with a model that included the effects of animal age and latency to touch the ball as covariates, and the animal as a random effect.

The parameter play (yes or no) was analyzed with Proc Logistic, modeled for the probability of the animal “play = yes” and with latency to touch as a covariate.

As the human approach test depended on the cooperation of the animals, it was not possible to obtain records from all of the animals on every day of the test, therefore only animals with 5, 4, 3, or 2 records were considered. We obtained results from 17 animals that performed the test more than 2 times, 9 animals that performed the test once, and 3 animals that never came to the feeding trough to perform the test.

Finally, a chi-square test of independence was performed between the distance to the trough and the type of exit from the chute, in which the distance to the trough was classified into only three classes as no animal was touched (<1 m, 1 to 2 m, and 2 to 3 m), and the type of exit from the chute into two classes (pace and trot, or gallop).

## 3. Results and Discussion

Data from 29 Mertolenga young bulls from 16 farms on six different dates were used. These animals started the test with an average of 321.5 ± 30.3 days of age and 320.1 ± 44.6 kg of weight, and finished the test with an average of 426.5 ± 30.3 days and 445.1 ± 55.1 kg.

According to Table 3, when evaluating the exit from the chute it can be seen that the animals averaged between pace (1) and trot (2). After leaving the chute, while in the area without the novelty element, they were active on average of 35.5 s. Once the ball was introduced to the park, the latency to touch the ball was 132.8 ± 95.0 s, the number of touches on the ball ranged from 0 to 9 touches, and most of the animals did not play with the ball (88%).

The average flight distance was 1.70 (in a scale from 1 to 3) and none of the animals were touched.

In Table 4 it can be seen that:

- the exit from the chute was significantly influenced by the effect of the farm where the animal came from, the age of the animal, and its weight.
- the activity of the animal during acclimatization to the space was significantly influenced by the animal itself, by the farm of origin, by the operator, and by the type of exit from the chute.
- the latency to touch the ball was significantly influenced by the animal, its farm of origin, and its type of exit from the chute.
- the number of touches on the ball was significantly influenced by the age of the animal and the latency to touch.
- the play behavior was significantly influenced by the latency to touch the ball.
- the distance from the operator to the feed trough in the human approach test was not significantly associated with the exit from the chute, with an error of 7% (*p* = 0.071).

It would be interesting to find a significant correlation between the exit from the chute and the distance to the trough, in order to be able to correlate the two behavior tests performed. However, the results showed a non-significant positive correlation (0.034; *p* = 0.81).

Table 5 shows significant correlations between the animal’s exit from the chute and its weight and age.

**Scheme 1:**
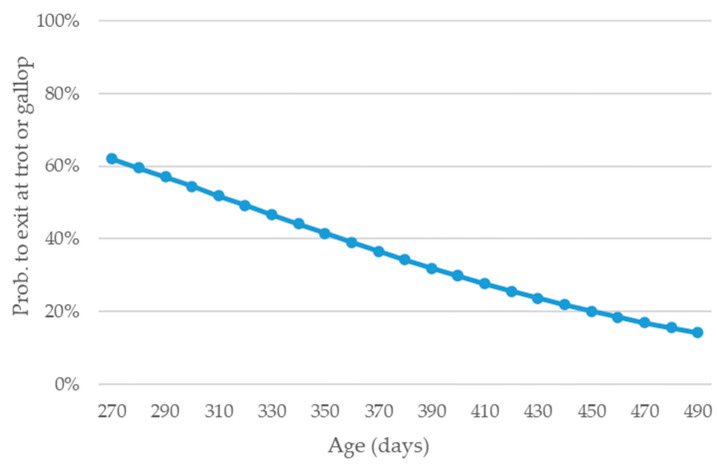
Effect of age of Mertolenga young bulls on the probability to exit the chute at trot or gallop.

As for the exit from the chute analysis, it was possible to observe that the older the animals, the less likely they were to exit the chute at trot or gallop, in other words, the greater the tendency to exit the chute more calmly, meaning that they would leave at pace (Scheme 1). These results are in agreement with those demonstrated by [14].

There were significant statistical differences on the exit of the chute between an animal leaving at pace and an animal leaving at gallop, as well as between an animal exiting at trot and an animal leaving at gallop (*p* < 0.01).

According to [18], chute exit speed tends to increase with age. In addition, animals with faster chute exits have been shown to have lower growth rates, worse carcasses, a weaker immune system, and higher cortisol levels when handling them [14]. Not only are they more difficult to handle and dangerous for keepers, they can also influence the behavior of the other animals around them. However, in our study we found that older animals were more likely to leave the chute walking (Scheme 1).

**Scheme 2:**
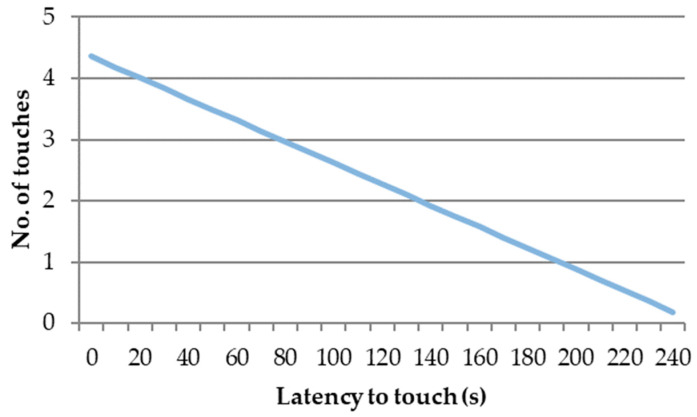
Effect of touch latency on the number of touches.

**Scheme 3:**
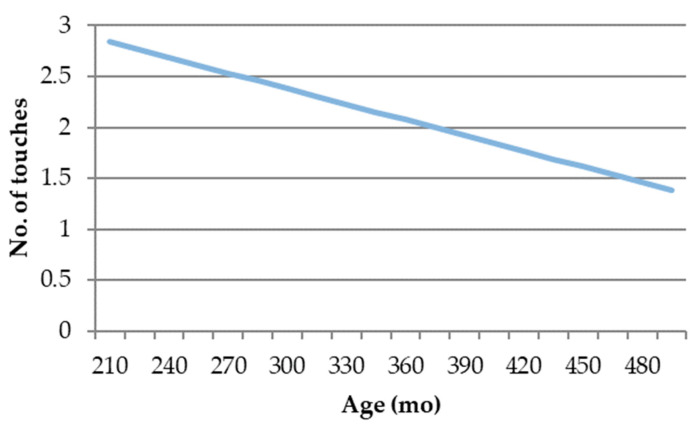
Effect of age on the number of touches.

Regarding the analysis of the latency to touch, the longer the animal took to touch the ball, the fewer times it touched the ball (Scheme 2). At times, this happened with very frightened animals that would leave the chute very quickly, and when the ball was placed in the park as a novelty factor, they would immediately invest on it. Meanwhile, calmer animals sometimes took a little longer to touch the ball, but then proceeded to play with it. As they became older, the number of touches also decreased (Scheme 3), perhaps also due to the habituation factor of the test. This lack of consistency in behavior between the tests was in agreement with [15], who also stated that the animals’ response to the novelty test may be due to the type of object used; in our study the same colored Pilates-ball was used.

**Scheme 4:**
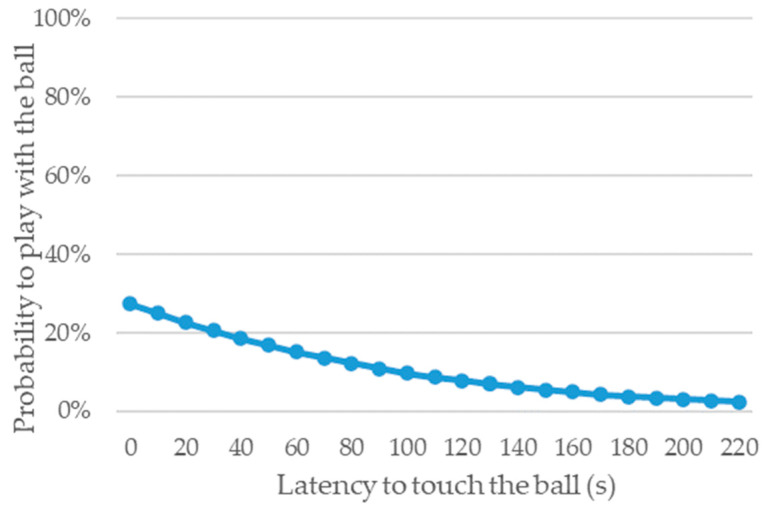
Effect of latency to touch the ball on the probability of the animal playing with the ball.

According to [13], habituation is common and a possible explanation for these results. In their study, which was conducted three times with a three to four-week interval, a decrease in animal interactions with a new object was observed. Scheme 4 shows that the longer the animal takes to touch the ball, the less likely it is to play with it. That is, the older the animal, the greater the latency to touch the ball and the fewer touches it makes to the ball (Scheme 2 and Scheme 3), with a consequent reduction in the probability of playing with it.

**Scheme 5:**
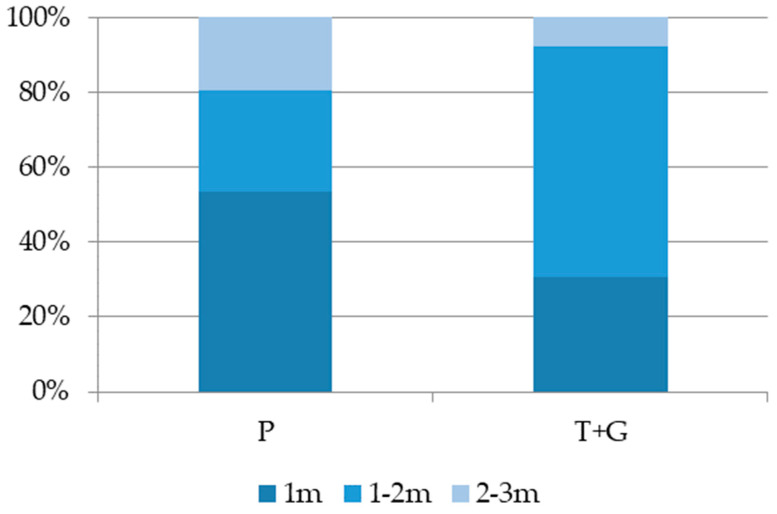
Distance from the operator to the feed trough in the human approach test according to the type of exit from the chute. Subtitle: P—pace, T+G—trot + gallop.

As for the human approach test, animals that left the chute more calmly, at pace, allowed themselves to be approached more than the animals that left the chute at trot or gallop (Scheme 5), as also mentioned by [15].

**Scheme 6:**
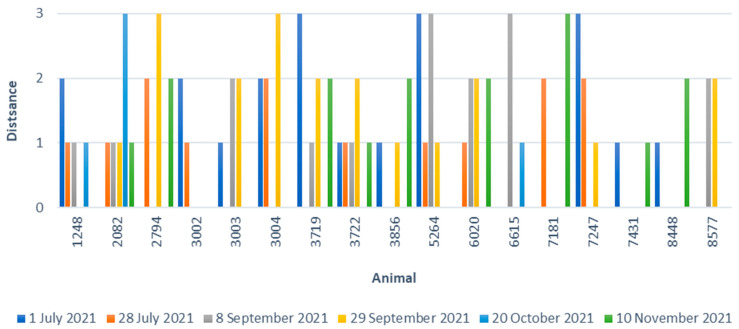
Results of the human approach test on some of the animals over time. Subtitle: 0—touched the animal, 1—until 1 m from the feed trough, 2—from 1 to 2 m from the feed trough, 3—from 2 to 3 m from the feed trough.

According to [3], the flight distance test performed at the feed trough was not affected by the age of the animals, and decreased significantly throughout the repetition of the test. These results were not observed in our study (only in animal 7247), and there was a great variability of results obtained for each animal (Scheme 6). As mentioned previously, not all animals performed the test (3 animals), and curiously, two of these three animals came from the same farm and always left the chute at a gallop pace—these were probably animals with fear/distrust issues. It is also suggested that the results of this human approach test were influenced by the animals surrounding the animal performing the test. Although we always endeavored to apply the test only to animals relatively isolated, this was not always possible.

## 4. Conclusions

The aim of this work was partially achieved. We managed to evaluate all the behaviors in question and obtain results that were interesting, and showed different responses. There was great variability in the animals’ responses in both tests, likely because the animals came from different farms and therefore had different experiences, but may also be due to genetic predisposition.

There was no evidence that the tests’ results were related to each other in any of our animals. It is necessary to continue data collection by performing behavioral tests with as many animals as possible, in order to understand all the factors involved including environmental and genetic factors, and even possible habituation to the tests over time.

The importance of animal welfare is being increasingly recognized. The parameters studied are related not only to management and ease of handling, but also to productivity, to products’ quality, and to profitability, as previously mentioned. Hence, there is a need to implement selection programs that take into account criteria such as the temperament of the animals. This could lead to the selection of calmer animals, which in turn tend to be more efficient.

We believe that these behavior tests should be further studied on potential sires, so as to increase docility and manageability of autochthonous beef breeds.

## Figures and Tables

**Table 1:** Heritability estimates for some behavioral tests in different beef cattle breeds.

Reference	Breed and Sample	Age at Test	Evaluated Measure	Heritability ± SE
[4]	Limousine (65)	8 months	Flight distance: Response to human approach (1–6)	0.17 ± 0.07–0.09
[5]	Angus (706) Charolais (556) Hereford (697) Limousine (424) Simmental (667)	5–11 months	Chute exit (1–4)	0.20 ± 0.08 0.25 ± 0.10 0.36 ± 0.06 0.11 ± 0.07 0.28 ± 0.07
[6]	Bos taurus (130)	6–12 months	“Habituation” to weighing on scale (difference between two repetitions of the test)	0.36 0.46

Adapted from [7].

**Table 2:** Distribution of animals among the different farms.

Farm	No. of Animals	Starting Age ( $\bar{X}$ Days)
A	1	265
B	1	287
C	2	303.5
D	2	292.5
E	1	281
F	2	302
G	2	267.5
H	2	293.5
I	2	287
J	1	367
K	2	246.5
L	4	337
M	2	348
N	2	265.5
O	2	287
P	1	273

**Table 3:** Descriptive statistics of some of the parameters evaluated.

Variable	N	Mean	SD	CV (%)	Min.	Max.
Chute exit (1–3)	159	1.55	0.74	47.7%	1	3
Activity (s)	146	35.50	13.85	39.0%	6	60
Latency to touch (s)	152	132.84	94.96	71.5%	2	240
No. of touches	156	2.10	2.34	111.4%	0	9
Play behavior	159	0.12	0.33	275.0%	0	1
Distance to the feed trough (0–3)	61	1.70	0.74	43.5%	1	3

Subtitle: Chute exit: 1—pace, 2—trot, 3—gallop; Distance to the feed trough: 0—touched the animal, 1—until 1 m from the feed trough, 2—from 1 to 2 m from the feed trough, 3—from 2 to 3 m from the feed trough; N—number of records obtained in each test.

**Table 4:** Summary of the results of the analysis on the different behaviors according to various models.

Behavior	SM		A		LT		NT		B		DM	
**Factors**	df	F	df	F	df	F	df	F	df	χ2	df	χ2
**Weight**	1	8.19 **										
**Farm**	10	2.53 **	10	2.38 *	15	4.11 **						
**Age**	1	4.78 *					121	5.06 *				
**LT**							121	154.55 **	2	9.24 **		
**Animal**				2.29 * Z		2.17 * Z						
**Op**			111	2.73 *								
**SM**			111	21.91 **	121	5.87 **					2	5.28+

Subtitle: SM—chute exit; A—activity; LT—latency to touch the ball; NT—number of touches; B—play behavior; DM—distance to the feed trough; df—degrees of freedom; F—value of F; X^2^—value of Qui-square test; Z—value of Z; Op—operator; ns—not significant (*p* > 0.05); * significant for *p* < 0.05; ** significant for *p* < 0.01; + significant for 0.05 < *p* < 0.1 (*p* = 0.07). 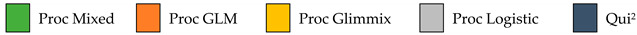

**Table 5:** Pearson correlation coefficients for some of the parameters analyzed.

	Chute Exit
**Weight**	−0.19
	(*p* < 0.05)
**Age**	−0.27
	(*p* < 0.01)

## Data Availability

The data that support the findings of this study are available from the corresponding author, A.V., upon reasonable request.
